# Hypomethylation and expression of *BEX2, IGSF4 *and *TIMP3 *indicative of *MLL *translocations in Acute Myeloid Leukemia

**DOI:** 10.1186/1476-4598-8-86

**Published:** 2009-10-16

**Authors:** Sonja Röhrs, Wilhelm G Dirks, Claus Meyer, Rolf Marschalek, Michaela Scherr, Robert Slany, Andrew Wallace, Hans G Drexler, Hilmar Quentmeier

**Affiliations:** 1 DSMZ-German Collection of Microorganisms and Cell Cultures, Braunschweig, Germany; 2 Institute of Pharmaceutical Biology/Diagnostic Centre of Acute Leukaemia, Goethe University, Frankfurt, Germany; 3 Department of Hematology, Hemostasis, Oncology and Stem Cell Transplantation, Medical School Hannover, Hannover, Germany; 4 Department of Genetics, Friedrich-Alexander University, Erlangen, Germany; 5 Department of Medical Genetics, St Mary's Hospital, Manchester, UK

## Abstract

**Background:**

Translocations of the *Mixed Lineage Leukemia *(*MLL*) gene occur in a subset (5%) of acute myeloid leukemias (AML), and in mixed phenotype acute leukemias in infancy - a disease with extremely poor prognosis. Animal model systems show that *MLL *gain of function mutations may contribute to leukemogenesis. Wild-type (wt) *MLL *possesses histone methyltransferase activity and functions at the level of chromatin organization by affecting the expression of specific target genes. While numerous MLL fusion proteins exert a diverse array of functions, they ultimately serve to induce transcription of specific genes. Hence, acute lymphoblastic leukemias (ALL) with *MLL *mutations (*MLL*mu) exhibit characteristic gene expression profiles including high-level expression of *HOXA *cluster genes. Here, we aimed to relate *MLL *mutational status and tumor suppressor gene (TSG) methylation/expression in acute leukemia cell lines.

**Results:**

Using MS-MLPA (methylation-specific multiplex ligation-dependent probe amplification assay), methylation of 24 different TSG was analyzed in 28 *MLL*mu and *MLL*wt acute leukemia cell lines. On average, 1.8/24 TSG were methylated in *MLL*mu AML cells, while 6.2/24 TSG were methylated in *MLL*wt AML cells. Hypomethylation and expression of the TSG *BEX2, IGSF4 *and *TIMP3 *turned out to be characteristic of *MLL*mu AML cell lines. *MLL*wt AML cell lines displayed hypermethylated TSG promoters resulting in transcriptional silencing. Demethylating agents and inhibitors of histone deacetylases restored expression of *BEX2, IGSF4 *and *TIMP3*, confirming epigenetic silencing of these genes in *MLL*wt cells. The positive correlation between *MLL *translocation, TSG hypomethylation and expression suggested that *MLL *fusion proteins were responsible for dysregulation of TSG expression in *MLL*mu cells. This concept was supported by our observation that *Bex2 *mRNA levels in *MLL-ENL *transgenic mouse cell lines required expression of the *MLL *fusion gene.

**Conclusion:**

These results suggest that the conspicuous expression of the TSG *BEX2, IGSF4 *and *TIMP3 *in *MLL*mu AML cell lines is the consequence of altered epigenetic properties of *MLL *fusion proteins.

## Background

Translocations of the *Mixed Lineage Leukemia *(*MLL*) gene occur in a subset of acute leukemias. The correlation between *MLL *translocations and expression of specific gene clusters is so evident that "mixed lineage leukemia", originally applied to biphenotypic acute leukemia cells, is now used to describe the *MLL *mutant (*MLL*mu) acute leukemias [1]. High expression levels of a set of *HOXA *cluster genes are characteristic of *MLL *mutations in primary acute lymphoblastic leukemia (ALL) cells, and in *MLL*mu ALL cell lines [1,2]. For acute myeloid leukemia (AML) cell lines, a similar correlation exists between *MLL *translocations and expression of the gene *brain expressed X-linked 2 *(*BEX2*, formerly called *BEX1*) [3]. In healthy people, *BEX2 *is expressed in the brain and, more weakly, in pancreas and testis, but not in hematopoetic cells [3,4]. In leukemia cell lines, we found *BEX2 *expression to be restricted to *MLL*mu AML. *MLL *wild-type (*MLL*wt) AML and ALL cell lines and, notably, also *MLL*mu ALL cell lines do not transcribe this gene, suggesting that *BEX2 *expression might be a diagnostic marker for *MLL*mu AML [3].

Several lines of evidence indicate that epigenetic mechanisms are responsible for the regulation of *BEX2 *expression: (i) the *BEX2 *promoter is methylated in *MLL*wt and unmethylated in *MLL*mu AML cell lines, thus demonstrating an inverse correlation between gene expression and promoter methylation [5]; (ii) demethylating agents and inhibitors of histone deacetylases (HDAC) induce *BEX2 *expression in *MLL*wt cells [5]; (iii) chromatin immunoprecipitation experiments show that histone acetylation plays a role in *BEX2 *regulation: immunoprecipitation of acetylated histone H3 coprecipitates chromatin from the 5' region of *BEX2 *in *MLL*mu, but not in *MLL*wt cells [5].

*BEX1 *and *BEX2 *have recently been described as epigenetically controlled candidate tumor suppressor genes (TSG) in malignant glioma [6]. Promoter hypermethylation of TSG is often seen in malignant diseases and, according to a widely held view, contributes to the rise of malignant cell clones by restraining tumor suppressor gene expression [7]. Moreover, unique profiles of hypermethylated CpG islands have been described which are characteristic of different neoplasias [8,9].

We applied a multiplex methylation detection assay to find out whether the connection between the *MLL *mutational status and promoter methylation is unique to *BEX2 *or if this correlation applies to other TSG as well. Results show that *MLL*wt AML cell lines exhibit a higher propensity for TSG promoter hypermethylation than *MLL*mu cell lines. This is especially true for *Immunoglobulin superfamily member 4 *(*IGSF4/CADM1), Retinoic acid receptor beta (RARB) *and *Tissue inhibitor of matrix metalloproteinase 3 *(*TIMP3*), all with *MLL*-dependent methylation profiles resembling *BEX2*. According to methylation-specific PCR (MSP), primary AML cells without rearrangement of the *MLL *gene also show a preference for TSG hypermethylation.

Our experimental results suggest that MLLmu proteins enhance the expression of distinct TSG and that this might be the consequence of altered epigenetic regulatory mechanisms of the fusion proteins.

## Results and Discussion

### Methylation patterns of TSG differ in *MLL*mu and *MLL*wt cell lines

Hypermethylation of CpG islands in the promoter regions of TSG occurs widely in malignancy, resulting in transcriptional inactivation which promotes cancerogenesis [7]. Unique profiles of hypermethylated CpG islands have been described, being characteristic of different neoplasias [8,9]. *BEX2 *is a newly described TSG, which is silenced by hypermethylation in malignant glioma [6]. With leukemia/lymphoma cell lines as model systems, we have shown that *BEX2 *hypomethylation and gene expression typifies *MLL*mu AML [3]. *BEX2 *silencing in *MLL*wt cells is the result of epigenetic mechanisms like promoter methylation and histone deacetylation [3,5]. Here, we set out to elucidate whether the described correlation between promoter hypomethylation and genetically rearranged *MLL *is peculiar to *BEX2*, or whether other TSG display similar features.

**Figure 1:**
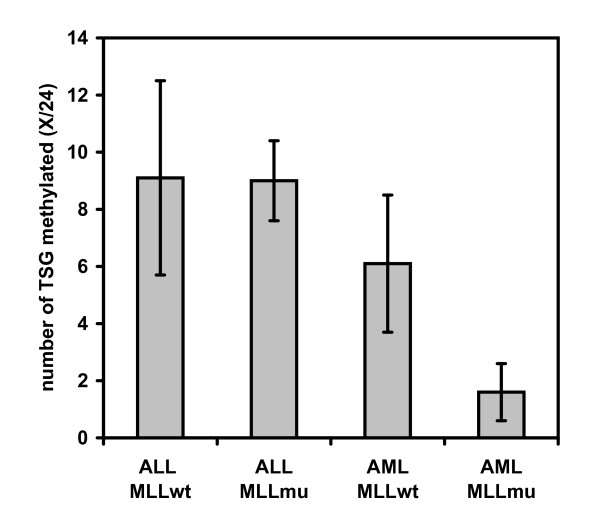
**Low tendency for TSG methylation in *MLL *mu AML cell lines**. Methylation status of 24 TSG was determined by MS-MLPA (see also: Additional file 1). Mean numbers of methylated TSG and standard deviations in the different groups of leukemia cell lines are shown. Note that *MLL*mu AML, but not *MLL*mu ALL cell lines show reduced TSG methylation.

Methylation-specific multiplex ligation-dependent probe amplification (MS-MLPA) was performed to assess the methylation status of 24 TSG in 28 acute leukemia cell lines, 50% with *MLL *translocations (see Additional file 1). ALL-derived cell lines showed a higher percentage of methylated TSG than AML cell lines (Fig. 1). The mean number of methylated TSG in ALL cell lines was independent of *MLL *mutational status (Fig. 1). In contrast, 25% of all TSG analyzed were methylated in *MLL*wt, but only 7% in *MLL*mu AML cell lines (Fig. 1). Provided that the TSG tested are representative, *MLL*mu AML cell lines display a significantly lower tendency to TSG hypermethylation than *MLL*wt cell lines.

### Methylation status of *BEX2, IGSF4, RARB *and *TIMP3 *is indicative of *MLL*mu AML

Expression array analysis allows classification of tumors, including the discrimination of acute leukemias with/without *MLL *translocations [1]. Likewise, profiles of promoter hypermethylation are unique for different types of cancer and have also been used to identify lymphomatous entities [8-12]. We evaluated our MS-MLPA data to find out whether *MLL*mu and *MLL*wt cell lines could be distinguished via their respective TSG methylation patterns (see Additional file 1). In addition to the 24 TSG included in this assay, we tested methylation of *BEX2 *by a methylation-sensitive DNA restriction- and quantitative real-time PCR-assay.

**Figure 2:**
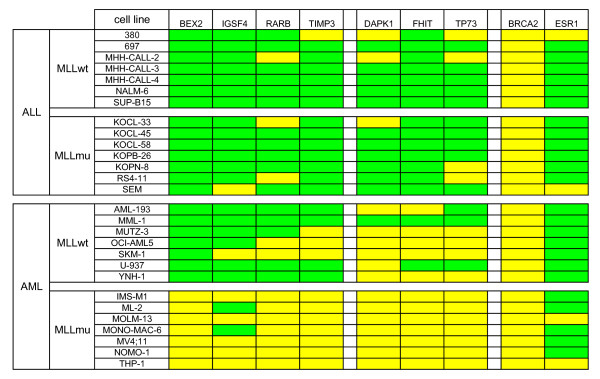
**TSG methylation predictive for classification of acute leukemia cell lines**. Methylation was assessed by MS-MLPA (see also Additional file 1) or by DNA digestion with methylation-sensitive *Hha*I plus promoter-specific quantitative real-time PCR (for analysis of *BEX2*). Green: methylation level ≥ 10%; yellow: methylation level < 10%. *BEX2, IGSF4, RARB *and *TIMP3 *are methylated in ALL and in *MLL*wt AML, but not in *MLL*mu AML. This correlation is statistically significant with p-values < 0.05 for *BEX2*, *RARB *and *TIMP3 *and with a p-value of 0.051 for *IGSF4 *(Fisher's exact test). Methylation analysis of *DAPK1, FHIT *and *TP73 *discriminates between ALL and AML cell lines (p-values ≤ 0.01, Fisher's exact test). Other TSG (e.g. *BRCA2 *and *ESR1*) are methylated or unmethylated in the majority of cell lines with no preference for subtype.

Promoter methylation of TSG *BEX2*, *IGSF4*, *RARB *and *TIMP3 *distinguished *MLL*mu and *MLL*wt AML: 0/7 *MLL*mu and 6/7 *MLL*wt AML cell lines exhibited methylation of at least two of these four TSG (p-value = 0.002, Fisher's exact test) (Fig. 2). Neither these four genes nor any other set of TSG analyzed distinguished between *MLL*mu and *MLL*wt ALL cell lines, suggesting that a myeloid gene expression background was obligatory for the *MLL*-specific methylation pattern (see Fig. 2, Additional file 1).

Most of the 24 TSG analyzed did not allow the histological origin of the cells to be inferred, being either methylated (e.g. *ESR1*) or unmethylated (e.g. *BRCA2*) in the majority of cell lines (see Fig. 2, Additional file 1). Furthermore, deletions, notably those of the TSG *CDKN2A *and *CDKN2B*, occurred in ALL as well as in AML cell lines (see Additional file 1). However, the methylation pattern of three genes (*DAPK1*, *FHIT*, *TP73*) was highly characteristic (p-values = 0.01, Fisher's exact test) of either ALL or AML (Fig. 2). These results show that analysis of TSG methylation may also be used to recognize distinct leukemic entities. Noteworthy in the context of our study, methylation patterns of *BEX2, IGSF4, RARB *and *TIMP3 *allowed discrimination between *MLL*mu and wt AML cell lines (Fig. 2).

MS-MLPA is a screening technique and obtained results rely on the analysis of one single CpG-site in the promoter region. To verify the validity of the methylation status as determined by MS-MLPA or *HhaI*-restriction-sensitive PCR assay (for *BEX2*), we performed MSP after bisulfite conversion of DNA for *BEX2, IGSF4 and TIMP3 *(see Additional file 2). The respective CpGs tested by MSP and MS-MLPA were located at different promoter sites. The accuracy of MS-MLPA/*Hha*I-restriction-sensitive PCR to predict the methylation status of these three TSG as determined by MSP was reassuringly high (83%), supporting the validity of MS-MLPA for use in our screening.

In conclusion, most *MLL*wt cell lines show hypermethylation of *BEX2, IGSF4 *and *TIMP3*, while *MLL*mu cell lines are preferentially unmethylated.

### Methylation of tumor suppressor genes in primary *MLL*mu and *MLL*wt AML cells

CpG island hypermethylation occurs more often in cell lines than in primary tumor cells [11,13]. Controversial opinions exist on the question whether or not cell lines, including leukemia cell lines, show the same methylation profiles as the analogous primary cells [13-15]. To investigate the possible concordance of TSG methylation in cell lines and primary cells, we tested samples from *MLL*mu and *MLL*wt AML patients for TSG methylation. MS-MLPA assay detected low numbers of methylated TSG (mean 0.5/24 +/- 1.0 TSG) in primary AML cells, which is in accordance with earlier findings applying the same technique [16]. MSP has been shown to be a suitable and more sensitive technique for primary AML samples than MS-MLPA [17].

**Figure 3:**
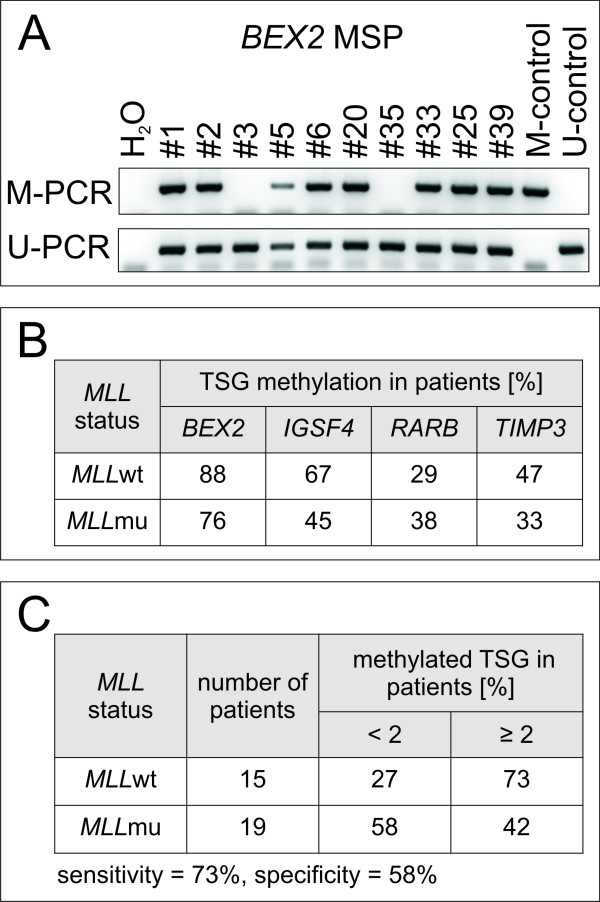
**TSG methylation in primary AML samples**. Methylation status was assessed by MSP for *BEX2*, *IGSF4*, *TIMP3 *and *RARB *in a panel of *MLL*wt and *MLL*mu AML patients. A) Representative results for *BEX2 *MSP are shown. Patients #1 - #17 carry *MLL*wt, patients #18 - #40 harbor *MLL *rearrangements (see Additional file 3). B) Summary of methylation data obtained by MSP in patients. Lower percentages of *MLL*mu patients than *MLL*wt patients show promoter hypermethylation of *BEX2, IGSF4 *and *TIMP3 *(see also Additional file 3). C) Note that methylation of two out of three TSG (*BEX2, IGSF4*, *TIMP3*) is more frequent in *MLL*wt patients than in *MLL*mu patients.

**Figure 4:**
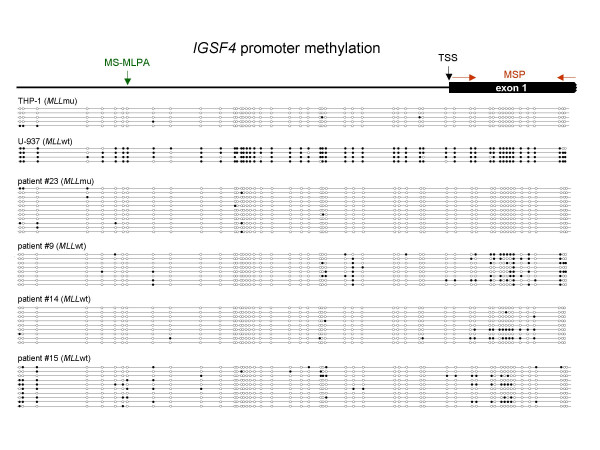
**Bisulfite sequencing of the *IGSF4 *promoter region**. The *IGSF4 *promoter region (559 bp, 52 CpG sites) was sequenced after bisulfite conversion of DNA in two cell lines and selected AML patients. CpGs are represented as open dots (unmethylated) or filled dots (methylated). In *MLL*wt AML patients and in the *MLL*wt cell line U-937 clones with CpG methylation next to the transcriptional start site (TSS) were detected whereas no methylation was detectable in the *MLL*mu patient analyzed and in the *MLL*mu cell line THP-1. Note that CpG sites analyzed by the MS-MLPA probe (green) and MSP primers (red) are not identical.

Therefore, we performed MSP after bisulfite conversion of DNA and detected methylated and unmethylated TSG promoter sequences using primers specific for *BEX2, IGSF4, RARB *and *TIMP3*, the most informative genes in our system (Fig. 3). For *BEX2*, *IGSF4 *and *TIMP3*, MSP indicated a trend towards an association between promoter methylation and *MLL*wt status (see Fig. 3B and Additional file 3). This correlation could not be observed for *RARB*, rendering this gene less interesting for our study. Bisulfite sequencing of the *IGSF4 *promoter region confirmed MSP results: *MLL*wt AML patients (#9, #14 and #15) and *MLL*wt cell line U-937 harbored clones with CpG methylation adjacent to the transcriptional start site of *IGSF4*, unlike *MLL*mu patient #23 and cell line THP-1 (see Fig. 4, Additional file 3).

Using methylation of at least two out of the three relevant genes (*BEX2, IGSF4 *and *TIMP3*) as classifier for the *MLL *mutational status, the true positive identification rate (sensitivity) for *MLL*mu samples was 73%, with a specificity of 58%, supporting the conclusion that methylation of *BEX2, IGSF4 *and *TIMP3 *is more frequent in *MLL*wt than in *MLL*mu patients (Fig. 3C). Testing a larger cohort of patients will be necessary to confirm a statistically significant positive correlation between *MLL *mutational status and TSG methylation.

### Inverse correlation of TSG methylation and expression

**Figure 5:**
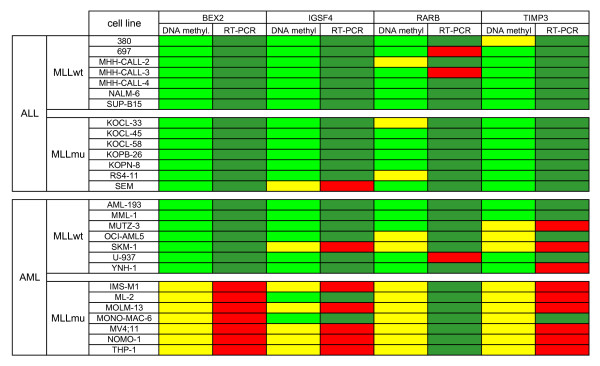
**Inverse correlation between TSG methylation and expression**. Hypomethylation (yellow) parallels RNA expression (red) of *BEX2, IGSF4 *and *TIMP3 *in *MLL*mu AML. *MLL*wt AML cell lines show hypermethylation (light green) and transcriptional silencing (green) of these. In contrast, *RARB *expression is low (green) even in cell lines that carry the unmethylated promoter (yellow). Excluding *RARB*, TSG hypermethylation inversely correlated with gene transcription in 94% of the cases. TSG methylation status is shown as determined by MS-MLPA (see Additional file 1). Gene expression was assessed in triplicates by quantitative real-time PCR (RT-PCR) analysis. For *BEX2, IGSF4 *and *TIMP3*, cell line IMS-M1 was applied as calibrator; for *RARB*, expression levels in cell line 697 were set as 1. Cell lines that reached relative expression levels ≥ 0.05 were considered positive (red).

Hypermethylation of CpG islands in the promoter regions of TSG generally leads to the silencing of the respective genes [18,19]. To test whether *BEX2*, *IGSF4*, *RARB *and *TIMP3 *were epigenetically regulated, we analyzed mRNA expression of these genes in cell lines by quantitative real-time PCR. Figure 5 shows that three of the four genes analyzed (*BEX2*, *IGSF4 *and *TIMP3*) demonstrated an inverse correlation between TSG promoter methylation and gene expression consistent with epigenetic gene regulation. Predictive value for true methylation of these genes was 0.93, and accuracy 0.94. In contrast, *RARB *remained silent in most cell lines, independent of methylation status (Fig. 5). A similar result has been described for *RARA*: primary AML cells show low *RARA2 *expression although the promoter is not methylated confirming that regulation of gene transcription is a multi-factor process, not solely reliant on promoter methylation/demethylation [20]. Thus, (i) *MLL*mu AML cell lines express higher levels *of BEX2, IGSF4 *and *TIMP3 *than *MLL*wt cell lines, and (ii) these three TSG appear to be regulated by epigenetic mechanisms.

*RARB *was excluded from further studies, as we neither observed an inverse correlation between *RARB *promoter methylation and gene expression in cell lines nor did we see a positive correlation between *MLL*mu and *RARB *hypomethylation in primary AML cells.

According to our findings, TSG hypomethylation and mRNA expression are characteristic features of *MLL*mu AML cells. This is in apparent conflict with the notion that MLL fusion proteins act as strong oncogenes, a view that is supported by the fact that *MLL*mu confers a dismal prognosis in AML. We hypothesize that *BEX2, IGSF4 *and *TIMP3 *are sentinel *MLL *fusion gene targets, their function being overridden by the strong oncogene *MLL*mu. A better understanding of how MLL fusion proteins impede TSG promoter methylation might help to illuminate a novel epigenetic role for *MLL*mu in leukemic cells.

### DNA demethylation and histone acetylation induce expression of TSG

To verify epigenetic regulation of *BEX2, IGSF4 *and *TIMP3*, we applied the DNA-demethylating agent 5-Aza-2'-deoxycytidine (Aza) and the HDAC inhibitor trichostatin A (TSA). Our previous results had shown that these agents induced expression of *BEX2 *in *MLL*wt AML cell lines [5]. We show here that Aza and TSA also triggered mRNA expression of *IGSF4 *and *TIMP3 *(Table 1). To confirm activity of TSA, histone H4K12 acetylation, a modification associated with active transcription, was analyzed (see Additional file 4). These results confirmed that epigenetic mechanisms are responsible for TSG silencing in *MLL*wt cell lines and suggest that TSG hypomethylation in *MLL*mu cell lines might be caused by alterations of epigenetic *MLL *functions as result of the various translocations.

**Table 1:** Effects of Aza and TSA on TSG expression in *MLL*wt AML cell lines.

**cell line**	**treatment**	**TSG**		
		
		***BEX2***	***IGSF4***	***TIMP3***
AML-193	TSA	-	-	**+**
	Aza	**++**	**++**	**+++**
	Aza + TSA	**++**	**++**	**+++**

SKM-1	TSA	**(+)**	**+**	**+**
	Aza	-	**(+)**	**+**
	Aza + TSA	**+**	**+**	**++**

U-937	TSA	-	**+**	**++**
	Aza	-	**+++**	**+++**
	Aza + TSA	-	**+++**	**+++**

Induction of TSG expression by Aza (5 μM, 4 d) and TSA (2 μM, 1 d) when compared to untreated control cells: - < 2-fold, + ≥ 2-fold; ++ ≥ 10-fold, +++ ≥ 100-fold gene expression. Expression levels were determined by quantitative real-time PCR. Note that cell line SKM-1 shows hypomethylation and expression of *IGSF4 *and *TIMP3 *(Fig. 5). Experiments were performed at least twice. Brackets: effect variable.

### *Bex2 *transcription depends on expression of *MLL-ENL*

**Figure 6:**
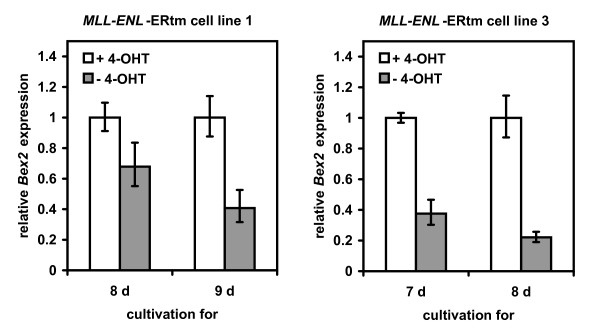
**MLL fusion proteins regulate *Bex2 *expression**. In presence of 4-OHT (100 nM) *MLL-ENL*-ERtm mouse cell lines expressed *MLL-ENL *and *Bex2*. Withdrawal of 4-OHT for seven to nine days induced downregulation of *MLL-ENL *and suppression of *Bex2 *transcription. *Bex2 *expression levels with standard deviations were determined in two different *MLL-ENL*-ERtm cell line clones by quantitative real-time PCR analysis.

To verify whether MLL fusion proteins were responsible for altered expression of TSG, we applied a mouse bone marrow cell line system that expresses *MLL-ENL *fused to the ligand-binding domain of the estrogen receptor. Removal of 4-hydroxytamoxifen (4-OHT) from the medium tethers MLL-ENL to a heat shock protein complex, thereby blocking the activation of the fusion protein [21]. Withdrawal of 4-OHT led to a marked decrease of *Bex2 *expression in this mouse cell system confirming that MLL fusion proteins serve to upregulate this gene (Fig. 6).

These results suggest that expression of TSG in *MLL*mu cell lines is attributable to the presence of MLL fusion proteins, raising the question whether the MLL fusion proteins directly target TSG promoters by blocking DNA methylation processes, or whether these effects are secondary.

It is difficult to attribute a specific oncogenic function to any single region of *MLL *fusion genes. Characteristic of the complexity of the situation is that many *MLL *translocations are reciprocal, and that both fusion genes are transcribed and may function oncogenically. However, *MLL-ENL *alone was sufficient to induce *Bex2 *in the transgene system indicating that the reciprocal fusion product was not required for regulation of this TSG. Furthermore, we observed TSG hypomethylation in various *MLL*mu cell lines with a variety of fusion partners. Together, these observations suggested that the inhibitory effect of *MLL *fusion genes on TSG promoter methylation was mediated by the N-terminal part of MLL and not by the fusion partner.

Both, the N-terminal and the C-terminal part of *MLL *have been implicated in transcriptional and/or epigenetic regulation. Located 3', the Su(var)3-9, enhancer-of-zeste, trithorax (SET) domain confers histone H3K4 methyltransferase activity to the protein [22]. However, histone H3K4 methylation is a marker for epigenetic activation and it appears unlikely that the loss of an activating function should lead to expression of TSG. Located upstream of the *MLL *breakpoint cluster region and retained in *MLL *fusion genes is the CXXC DNA methyltransferase homology domain, which binds CpG rich regions particularly when these are unmethylated [23]. This domain is essential for target gene recognition and is required for transformation by MLL fusion proteins [24]. Therefore, the CXXC domain might direct MLL to transcribed genes or possibly protect CpG islands against methylation. The N-terminal part of MLL has also been reported to bind menin [25]. MLL and menin are required for cellular transformation and both proteins cooperatively regulate expression of the genes p27^Kip1 ^and p18^Ink4c ^[25,26].

Thus, regulation of TSG expression and alterations of epigenetic functions of MLL fusion proteins are feasible consequences of *MLL *translocations, but whether the TSG hypomethylation in *MLL*mu cell lines described by us is the direct effect of MLL fusion proteins or whether the hypomethylation is rather an indirect consequence, remains to be resolved. For two reasons we favor the latter explanation: *MLL*mu ALL cell lines do not show the TSG methylation profile that is typical for *MLL*mu AML suggesting that additional, tissue-specific factors play a role in this context. Furthermore, ChIP-DNA mircroarrays did not identify *TIMP3 *as target of wild-type *MLL*, the other two genes not being tested [27]. Future studies will have to provide evidence whether the MLL fusion proteins show altered DNA binding specificities than the wild-type protein allowing access to the promoters of the named TSG.

## Conclusion

We describe the conspicuous expression of TSG *BEX2, IGSF4 *and *TIMP3 *in *MLLmu *AML cells. In *MLLwt *cell lines these genes are silenced by promoter methylation. Transcription could be reactivated by treatment with demethylating agents and HDAC inhibitors. In *MLLmu *AML cell lines, constitutive expression of *BEX2, IGSF4 *and *TIMP3 *was accompanied by promoter hypomethylation. Ectopic expression of *MLL-ENL *drove upregulation of *Bex2 *in a tamoxifen-inducible mouse model indicating that the *MLL *fusion genes were responsible for upregulation of TSG mRNA. Taken together, our results suggest that hypomethylation of TSG characteristic of *MLL*mu AML cells may be the consequence of *MLL *gene alterations, including elements responsible for epigenetic regulation.

## Methods

### Human cell lines

The continuous cell lines were either taken from the stock of the cell bank (DSMZ - German Collection of Microorganisms and Cell Cultures) or were generously provided by the original investigators. Detailed references and cultivation protocols have been described previously [28,29]. The following cell lines were tested for TSG methylation: (i) B-cell precursor (BCP) ALL-derived cell lines expressing *MLL*wt: 380, 697, MHH-CALL-2, MHH-CALL-3, MHH-CALL-4, NALM-6, SUP-B15; (ii) BCP ALL-derived cell lines with *MLL*mu: KOCL-33 t(11;19), KOCL-45 t(4;11), KOCL-58 t(4;11), KOPB-26 t(9;11), KOPN-8 t(11;19), RS4;11 t(4;11), SEM t(4;11); (iii) AML-derived cell lines with *MLL*wt: AML-193 (M5), MML-1 (M1), MUTZ-3 (M4), OCI-AML5 (M4), SKM-1 (M5), U-937 (M5), YNH-1 (M1); (iv) AML-derived cell lines with *MLL*mu: IMS-M1 (M5, t(9;11)), ML-2 (M4, t(6;11)), MOLM-13 (M5, t(9;11)), MONO-MAC-6 (M5, t(9;11)), MV4;11 (M5, t(4;11)), NOMO-1 (M5, t(9;11)), THP-1 (M5, t(9;11)).

### Human tissue samples

After informed consent was given, bone marrow or peripheral blood specimens were obtained during routine clinical assessment of 40 AML patients. The collection of patient samples for analysis of genetic changes was approved by the local ethics committee. DNA extraction was performed from unselected cells from bone marrow or peripheral blood. The *MLL*wt/mu status of primary samples was assessed with long-distance PCR analysis, as described previously [30].

### Methylation-specific multiplex ligation-dependent probe amplification assay

The MS-MLPA assay (ME001B; MRC-Holland, Amsterdam, Netherlands) simultaneously detects copy number changes and CpG methylation of the promoter regions of 24 different TSG. This semi-quantitative technique is based on digestion of DNA with the methylation-sensitive restriction enzyme *Hha*I (Fermentas, St. Leon-Rot, Germany) and a subsequent multiplex PCR followed by fragment analysis via capillary electrophoresis [31]. MS-MLPA data were analysed using a Microsoft Excel spreadsheet designed specifically for the ME001B assay. Levels of methylation were calculated by comparing the relative peak area of the *HhaI *digested ligation product with the corresponding ligation product from the undigested sample. Peak areas were normalised relative to neighbouring control ligation products prior to comparison as recommended by MRC-Holland. The spreadsheet for the analysis of the ME001B kit is freely available for download and use on the National Genetics Reference Laboratory website at . For assessment of *BEX2 *promoter methylation, *Hha*I-digested and undigested DNA was used as template for subsequent quantitative real-time PCR using SYBR GREEN PCR Master Mix (Applied Biosystems, Foster City, CA, USA) in a 7500 Applied Biosystems real-time PCR system. The sequence flanked by primer pair A lacked *Hha*I sites, and was used as endogenous control. Sequence amplified by primer pair B contained two *Hha*I sites. The percentage of non-cleaved and thus methylated template in comparison to the undigested sample was calculated using the ΔΔCt-method. *BEX2 *A forward: 5'-GGT TGG TGA GAA GGA GGG TG-3'; *BEX2 *A reverse: 5'-GAG ACA CGA GTG ACG ACT GCA-3'; *BEX2 *B forward: 5'-TGG AGA GGA CGG AGA TGA GTG-3'; *BEX2 *B reverse: 5'-CAC CCT CCT TCT CAC CAA CC-3'. Methylation was scored positive when the calculated methylation percentage was ≥ 10%.

### Methylation-specific polymerase chain reaction (MSP)

Bisulfite conversion of DNA was performed as described by the supplier (Active Motif, Rixensart, Belgium). For detecting TSG promoter methylation, we performed nested PCR with first round primers amplifying converted DNA independently of the methylation status (bisulfite-specific PCR), while second round primers for M- and U-PCR specifically recognized the methylated or unmethylated versions of the promoter. PCR products of the initial bisulfite-specific PCR were diluted 1:100 for subsequent M- and U-PCR. PCR conditions and primer sequences are listed in Additional file 5. Epitect PCR Control DNA (Qiagen, Hilden, Germany) was used as control for methylated and unmethylated templates.

### Bisulfite sequencing

To confirm methylation status of the *IGSF4 *promoter, DNA of cell lines and AML patients was bisulfite converted according to the manufacturer's instructions (Active Motif). Subsequently, amplification of the *IGSF4 *promoter region (559 bp) was performed using primers *IGSF4 *BSP fwd and *IGSF4 *BSP rev, specifically binding bisulfite converted DNA (for primer sequence and PCR conditions see Additional file 5). Resulting *IGSF4 *fragments were purified, cloned into pGEM-T Easy vector (Promega, Madison, WI, USA) and sequenced. Sequences were evaluated using BiQ Analyzer  and had to conform to at least 90% bisulfite conversion rate. In addition, identical clones were excluded from the analysis.

### Expression of tumor suppressor genes

Quantitative PCR was performed on a 7500 Applied Biosystems (Darmstadt, Germany) real-time PCR system using the manufacturer's protocol. RNA was prepared using the Trizol reagent (Invitrogen, Karlsruhe, Germany). For mRNA quantification, reverse transcription was performed using the SuperScript II reverse transcriptase kit (Invitrogen). TaqMan probes (Applied Biosystems) were used to quantify human *BEX2 *(Hs 00218464m1), *IGSF4 *(Hs 00204937m1), *RARB *(Hs 00233407m1) and *TIMP3 *(Hs 00927216m1) expression levels with *TBP *as endogenous control. Expression of mouse *Bex2 *was assessed using the SYBR GREEN PCR Master Mix (Applied Biosystems) with *Tbp *as internal control. *Bex2 *forward: 5'-GCG AGC GGG ACA GAT TGA C-3'; *Bex2 *reverse: 5'-TCC ATT TCT CCT GGG CCT ATC-3'. *Tbp *forward: 5'-ACC AGA ACA ACA GCC TTC CAC-3'; *Tbp *reverse: 5'-TGC CGT AAG GCA TCA TTG GAC-3'. Relative expression levels were calculated using the ΔΔCt-method.

### Induction of *MLL-ENL*

Mouse bone marrow cell lines *MLL-ENL*-ERtm 1 and 3 were maintained in RPMI 1640 medium supplemented with 10% FBS (Sigma, Taufkirchen, Germany), IL-3 (5 ng/ml), GM-CSF (5 ng/ml), IL-6 (5 ng/ml), SCF (50 ng/ml) and 4-OHT (100 nM). Murine cytokines were obtained from Richter-Helm (BioLogics, Hamburg, Germany) and 4-OHT was purchased from Sigma. 4-OHT releases MLL-ENL from a heat-shock protein complex thereby activating the fusion protein [21]. Note that MLL-ENL is essentially required for the continuous proliferation of this cell line.

### Treatment with demethylating agents and inhibitors of histone deacetylases

Aza (Sigma) was used to verify the effect of methylation on expression of TSG. The HDAC inhibitor TSA (Sigma) was applied for testing the role of histone acetylation for TSG expression. Cells were seeded at a cell density of 5 × 10^5 ^cells/ml, Aza was added at a final concentration of 5 μM. Control cells were treated with 0.05% DMSO. After 2 d, half of the medium was replenished with medium with/without Aza (5 μM). TSA (2 μM final concentration) was added for the last 24 h of cultivation. After 4 d, cells were harvested to prepare RNA and protein.

### Histone purification and Western blot analysis

Histones were purified according to the protocol of the supplier (Active Motif). Then, 3.3 μg or 10 μg protein were separated by electrophoresis (15% SDS gels) to detect histones and acetylated histones, respectively. Anti acetyl histone H4K12 antiserum was obtained from Biomol/Upstate (Hamburg, Germany), anti histone H4 monoclonal Ab was purchased from Abcam (Cambridge, United Kingdom). Specific bands on nitrocellulose membranes were visualized with the biotin/streptavidin-horseradish peroxidase system (Amersham, Freiburg, Germany) in combination with the "Renaissance Western Blot Chemoluminescence Reagent" protocol (DuPont, Bad Homburg, Germany).

## Competing interests

The authors declare that they have no competing interests.

## Authors' contributions

SR designed parts of the study and performed MS-MLPA, MSP analysis, sequencing of bisulfite-converted DNA and co-wrote the manuscript, WGD established MLPA, CM performed *MLL*wt and *MLL*mu analysis for primary AML samples, RM provided patient samples and gave good advice, MS performed knock-down experiments, RS provided MLL-ENL-ERtm mouse cell lines and gave good advice, AW established the spreadsheets for the evaluation of MS-MLPA experiments, HGD provided cell lines and critically read the manuscript, HQ designed the study and wrote the manuscript.

## Supplementary Material

Additional file 1 **Methylation status of 24 TSG in 28 acute leukemia cell lines according to MS-MLPA**. Shown is the promoter methylation status of TSG. Green: methylation ≥ 10%; yellow: methylation < 10%. TSG are arranged from left to right according to gene locus. For TSG *CDKN2A *and *CDKN2B*, ploidy status is indicated by numbers. Data shown are from one experiment per cell line. Reproducibility of MS-MLPA results was confirmed by up to four repetitions of analyses with selected cell lines. Click here for file

Additional file 2 **MSP analyses of *BEX2*, *IGSF4 *and *TIMP3 *in AML cell lines**. Methylation status of *BEX2*, *IGSF4 *and *TIMP3 *were determined in *MLL*wt and *MLL*mu AML cell lines by MSP to control methylation status as determined by MS-MLPA. Results for M- and U-PCR are shown as well as the methylation status according to MS-MLPA. Performance of MS-MLPA as a classification system for methylated or unmethylated TSG was evaluated using a confusion matrix. Overall, the results of the techniques were in good concordance with an accuracy of 0.83. In detail, accuracy was 0.86 for *BEX2*, 0.93 for *IGSF4 *and 0.71 for *TIMP3*. Click here for file

Additional file 3 **Methylation analyses in primary AML samples**. Results of promoter methylation analysis according to MSP of *BEX2, IGSF4, RARB *and *TIMP3 *are shown for *MLL*mu and *MLL*wt AML patients Click here for file

Additional file 4 **Activating histone modifications induced by TSA**. TSA effected acetylation of histone H4K12, as assessed by Western blot analysis. Aza treatment was not alone sufficient to induce histone H4K12 acetylation. *MLL*wt cell lines AML-193, SKM-1 and U-937 were treated with TSA (2 μM, 1 d), Aza (5 μM, 4 d) or a combination of both reagents. Click here for file

Additional file 5 **Primers for methylation-specific PCR**. Primers and reaction conditions for BSP and MSP are listed. Click here for file
