# Supplementary figures and images for: Hypomethylation and expression of BEX2, IGSF4 and TIMP3 indicative of MLL translocations in Acute Myeloid Leukemia
Source: Mol Cancer. 2009 Oct 16;8:86. doi: 10.1186/1476-4598-8-86 (PMC2770485; doi:10.1186/1476-4598-8-86)

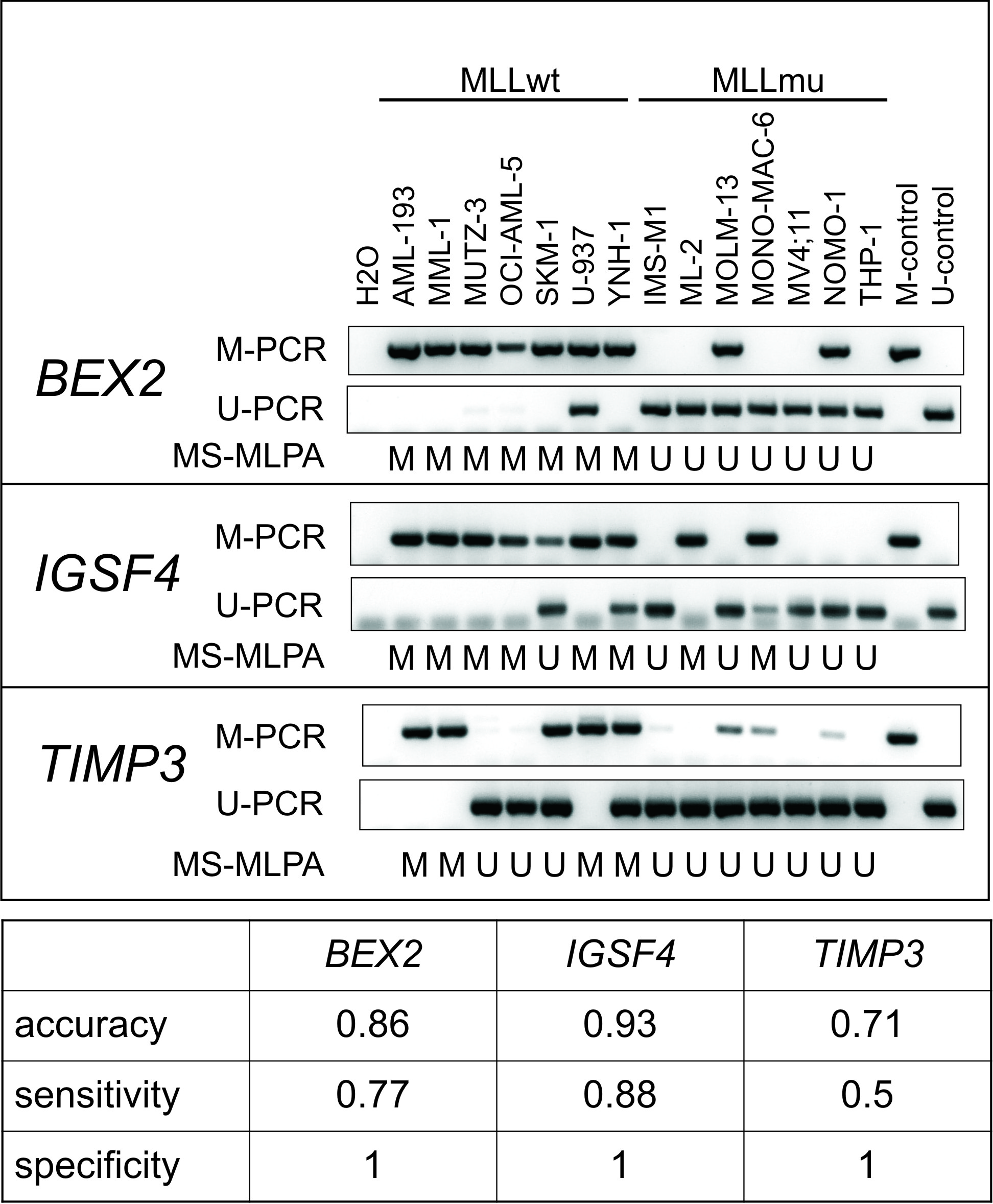

Supplement: Additional file 2 — MSP analyses of BEX2, IGSF4 and TIMP3 in AML cell lines. Methylation status of BEX2, IGSF4 and TIMP3 were determined in MLLwt and MLLmu AML cell lines by MSP to control methylation status as determined by MS-MLPA. Results for M- and U-PCR are shown as well as the methylation status according to MS-MLPA. Performance of MS-MLPA as a classification system for methylated or unmethylated TSG was evaluated using a confusion matrix. Overall, the results of the techniques were in good concordance with an accuracy of 0.83. In detail, accuracy was 0.86 for BEX2, 0.93 for IGSF4 and 0.71 for TIMP3. [file 1476-4598-8-86-S2.JPEG]

**acetyl. histone H4K12**

**histone H4**

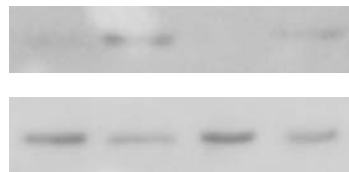

**AML-193**

**acetyl. histone H4K12**

**histone H4**

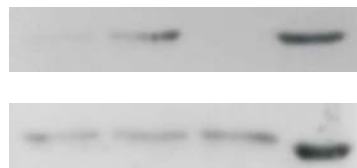

**SKM-1**

**acetyl. histone H4K12**

**histone H4**

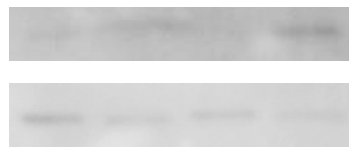

**U-937**

Supplement: Additional file 4 — Activating histone modifications induced by TSA. TSA effected acetylation of histone H4K12, as assessed by Western blot analysis. Aza treatment was not alone sufficient to induce histone H4K12 acetylation. MLLwt cell lines AML-193, SKM-1 and U-937 were treated with TSA (2 μM, 1 d), Aza (5 μM, 4 d) or a combination of both reagents. [file 1476-4598-8-86-S4.PDF]
