# Supplementary material for: Hypomethylation and expression of BEX2, IGSF4 and TIMP3 indicative of MLL translocations in Acute Myeloid Leukemia
Source: Mol Cancer. 2009 Oct 16;8:86. doi: 10.1186/1476-4598-8-86 (PMC2770485; doi:10.1186/1476-4598-8-86)
Supplement: Additional file 3 — Methylation analyses in primary AML samples. Results of promoter methylation analysis according to MSP of BEX2, IGSF4, RARB and TIMP3 are shown for MLLmu and MLLwt AML patients [file 1476-4598-8-86-S3.DOC]

Additional File 3: Methylation analyses in primary AML samples

| **MLL status** | **patient #** | **MSP** | | | |
| --- | --- | --- | --- | --- | --- |
|  |  | ***BEX2*** | ***IGSF4*** | ***RARB*** | ***TIMP3*** |
| MLLwt | 1 | + | - | - | - |
| MLLwt | 2 | + | - | n.d. | - |
| MLLwt | 3 | - | - | - | + |
| MLLwt | 4 | + | + | - | + |
| MLLwt | 5 | + | n.d. | n.d. | - |
| MLLwt | 6 | + | n.d. | n.d. | - |
| MLLwt | 7 | + | + | + | + |
| MLLwt | 8 | + | + | - | + |
| MLLwt | 9 | + | + | + | + |
| MLLwt | 10 | - | + | - | + |
| MLLwt | 11 | + | + | + | - |
| MLLwt | 12 | + | - | + | + |
| MLLwt | 13 | + | - | - | - |
| MLLwt | 14 | + | + | - | - |
| MLLwt | 15 | + | + | - | + |
| MLLwt | 16 | + | + | - | - |
| MLLwt | 17 | + | + | - | - |
| MLL-AF9 | 18 | + | + | + | - |
| MLL-AF9 | 19 | + | - | + | + |
| MLL-AF9 | 20 | + | - | - | - |
| MLL-AF9 | 21 | + | - | - | - |
| MLL-AF9 | 22 | + | + | - | - |
| MLL-AF9 | 23 | - | - | - | - |
| MLL-AF9 | 24 | - | - | + | + |
| MLL-AF9 | 25 | + | + | - | + |
| MLL-AF9 | 26 | + | - | - | - |
| MLL-AF9 | 27 | - | - | - | - |
| MLL-AF9 | 28 | + | n.d. | n.d. | n.d. |
| MLL-AF9 | 29 | - | + | + | - |
| MLL-AF9 | 30 | + | - | - | - |
| MLL-AF9 | 31 | n.d. | + | n.d. | + |
| MLL-AF9 | 32 | + | + | + | + |
| MLL-ELL | 33 | + | - | - | - |
| MLL-ELL | 34 | + | + | + | + |
| MLL-ENL | 35 | - | - | + | n.d. |
| MLL-ENL | 36 | + | + | - | + |
| MLL-AF6 | 37 | + | + | - | - |
| MLL-AF6 | 38 | + | - | + | - |
| MLL-AF10 | 39 | + | - | - | - |
| MLL-AF10 | 40 | n.d. | + | - | - |

Methylation status of TSG *BEX2*, *IGSF4*, *RARB* and *TIMP3* was determined by MSP: +: gene methylated, -: gene unmethylated, n.d.: not determined.
