# Supplementary material for: Hypomethylation and expression of BEX2, IGSF4 and TIMP3 indicative of MLL translocations in Acute Myeloid Leukemia
Source: Mol Cancer. 2009 Oct 16;8:86. doi: 10.1186/1476-4598-8-86 (PMC2770485; doi:10.1186/1476-4598-8-86)
Supplement: Additional file 5 — Primers for methylation-specific PCR. Primers and reaction conditions for BSP and MSP are listed. [file 1476-4598-8-86-S5.DOC]

Additional File 5: Primers for methylation-specific PCR

| **PCR** | **Annealing T** | **Primer** | **Sequence (5' → 3')** |
| --- | --- | --- | --- |
| Bisulfite-specific PCR BEX2 | 53°C | BEX2 BSP fwd | GAAAGAAGAAAAGGTTAAGGTT |
|  |  | BEX2 BSP rev | CCACCCCAAAAAACCACTATA |
| M-PCR BEX2 | 62°C | BEX2 M_fwd | AGTTTTCGGTGTAGTCGTTATTC |
|  |  | BEX2 M_rev | TACGCTATTCTACGCTCGTCG |
| U-PCR BEX2 | 62°C | BEX2 U_fwd | GTAGTTTTTGGTGTAGTTGTTATTT |
|  |  | BEX2 U_rev | CCTACACTATTCTACACTCATCA |
| Bisulfite-specific PCR IGSF4 | 53°C | IGSF4 BSP fwd | GGATTTTTTTAAGGGAGATTTTTTAG |
|  |  | IGSF4 BSP rev | CCCACACCTACCTATAAAAATCAA |
| M-PCR IGSF4 | 64°C | IGSF4 M_fwd | GCGAGTGTAGTGTTGTCGAGC |
|  |  | IGSF4 M_rev | CCTATAAAAATCAATACCGCGACG |
| U-PCR IGSF4 | 60°C | IGSF4 U_fwd | ATGGTGAGTGTAGTGTTGTTGAGT |
|  |  | IGSF4 U_rev | CTACCTATAAAAATCAATACCACAACA |
| Bisulfite-specific PCR RARB | 53°C | RARB BSP fwd | TTGTGGAATGGAAAGTTTTTTAAT |
|  |  | RARB BSP rev | AATCCCAAATTCTCCTTCCAAA |
| M-PCR RARB | 58°C | RARB M_fwd | GATTGGGATGTCGAGAACGC |
|  |  | RARB M_rev | AACCTTCCGAATACGTTCCGA |
| U-PCR RARB | 58°C | RARB U_fwd | AGGATTGGGATGTTGAGAATGT |
|  |  | RARB U_rev | AAAAACCTTCCAAATACATTCCAA |
| Bisulfite-specific PCR TIMP3 | 53°C | TIMP3 BSP fwd | TTTTGGTTTGGGTTAGAGATATTT |
|  |  | TIMP3 BSP rev | TCTACAAAACATCTTCCCCTCT |
| M-PCR TIMP3 | 56°C | TIMP3 M_fwd | AGTAGTAGTTTCGGTAGCGGC |
|  |  | TIMP3 M_rev | CCAAAAACGCTTACCGATATCG |
| U-PCR TIMP3 | 56°C | TIMP3 U_fwd | AGTAGTAGTTTTGGTAGTGGT |
|  |  | TIMP3 U_rev | CCAAAAACACTTACCAATATCA |
